# Supplementary material for: Effects of aerobic exercise on cognition and hippocampal volume in Alzheimer’s disease: study protocol of a randomized controlled trial (The FIT-AD trial)
Source: Trials. 2014 Oct 11;15:394. doi: 10.1186/1745-6215-15-394 (PMC4283145; doi:10.1186/1745-6215-15-394)
Supplement: Supplementary file 1 — Authors’ original file for figure 1 [file 13063_2014_2344_MOESM1_ESM.pdf]

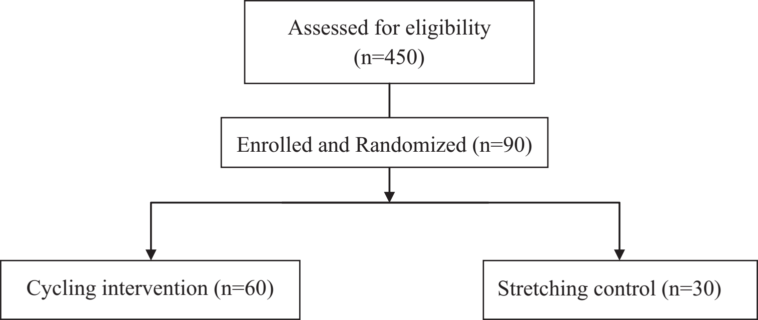
Assessed for eligibility
(n=450)
Enrolled and Randomized (n=90)
Cycling intervention (n=60)
Stretching control (n=30)
